# Supplementary material for: Visualising trends in dentition to lip mouth morphology using geometric morphometrics
Source: PLoS One. 2022 Sep 2;17(9):e0274127. doi: 10.1371/journal.pone.0274127 (PMC9439251; doi:10.1371/journal.pone.0274127)
Supplement: Attachment — Submitted filename: Response-to-Reviewers.docx [file pone.0274127.s003.docx]

Dear Reviewers,

We are most grateful for your positive feedback and invaluable advice. We have responded to each of your helpful comments and hope the article meets expectations for publication. Details on changes made are listed below.

Journal requirements:

1. Manuscript was updated to meet PLOS ONE's formatting requirements (e.g., updates to font size of headings).
2. Regarding participant consent, it is clarified that written consent was obtained by the institution.
3. The compiled raw data has been uploaded as Supporting Information files.
4. The independent ethics statement has been removed, with all comments on ethics maintained under Materials and Methods.
5. References checked to ensure they are complete and correct.

Comments to Author

Reviewer #1:

1. Population origin was self-prescribed – this is now noted in the second paragraph the Materials and Methods.
2. Text updated to state ‘Frankfurt/Frankfort Horizontal Plane’ where it is stated under Abstract and Materials and Methods.
3. Numbers less than 100 are now written as words (unless they are percentage or measurement values).
4. Under Materials and Methods a description to how the Frankfurt/Frankfort Horizontal Plane was achieved is given (see Pg. 6).
5. Page 8 line 2 – replaced ‘done’ with ‘carried out’.
6. The wireframes present a visual reference to how the lips tend to be positioned over the dentition. This visual information helps substantiate previous observations used in mouth approximation, and provides a visual guide that indicates how linear measurements are to be placed over the skull. The title has been adjusted to focus the paper as identifying trends in dentition to lip mouth morphology using geometric morphometrics.

Reviewer #2:

1. Title has been modified as recommended.
2. The statement "No existing geometric morphometric studies, however, has considered the spatial relationship between hard and soft facial tissues" has been updated to specifically state that this is in the instance of the South African population.
3. Inter-landmark distances were calculated using Pythagorean Theorem, see page 8 line 17 of paragraph.
4. Measurement error table is available in Table 3, Pg 10. Literature supporting the inter-observer measuring technique is provided in the Materials and Methods, on the last line of Pg 6.
5. Morpho J v.2.0; PAST v.4.03 – citation included (see Ref [29]).
6. Allometry is something we envisage exploring in more detail in a future study.
7. Figure 1 includes labels (a) and (b), and caption modified accordingly.
8. Labels PC1-PC4 have been included in the scatter plot.
9. Arrangement of discussion and conclusion have been updated as recommended. Conclusion has been transferred to the last paragraph of the discussion section while the last two paragraphs (Starting from: "From this study, we have largely identified shape, size and proportional changes related to demographic variables such as age, population, and sex...") have been transferred to the conclusion section.

Many thanks and kindest regards,

Tobias Houlton,

Nicolene Jooste,

Maryna Steyn,

Jason Hemingway
